# Fly Ash from Lignite Combustion as a Filler for Rubber Mixes—Part II: Chemical Valorisation of Fly Ash

**DOI:** 10.3390/ma15175979

**Published:** 2022-08-29

**Authors:** Wojciech Orczykowski, Dariusz M. Bieliński, Rafał Anyszka, Tomasz Gozdek, Katarzyna Klajn, Grzegorz Celichowski, Zbigniew Pędzich, Agnieszka Wojteczko

**Affiliations:** 1 BESTGUM POLSKA Ltd., Św. Barbary 3, 97-427 Rogowiec, Poland; 2 Institute of Polymer & Dye Technology, Lodz University of Technology, Stefanowskiego 16, 90-537 Lodz, Poland; 3 Department of Materials Technology and Chemistry, Faculty of Chemistry, University of Lodz, Pomorska 163, 90-236 Lodz, Poland; 4 Department of Ceramics and Refractories, AGH University of Science and Technology, Mickiewicza 30, 30-059 Cracow, Poland

**Keywords:** fractionated fly ash, silanization, characterization, rubber vulcanizates, mechanical properties

## Abstract

Fly ash (FA) fractions with a particle size of 63 µm < FA < 250 µm obtained by sieve fractionation were used as a partial carbon black (CB) replacement in a rubber mixture based on styrene-butadiene rubber (SBR). In order to improve the interactions at the interface between rubber and fractionated ash, at the stage of preparing the rubber mixtures, two different vinyl silanes were added to the system: Vinyltrimethoxysilane (U-611) or Vinyl-tris (2-methoxy-ethoxy) silane (LUVOMAXX VTMOEO DL50), silane with epoxy groups: 3-(glycidoxypropyl)trimethoxysilane (U-50) or sulfur functionalized silanes: containing sulfide bridges: Bis(triethoxysilylpropyl)polysulfide silane (Si-266) or mercapto groups: Mercaptopropyltrimethoxysilane (Dynaslan MTMO). The conducted research confirmed the effectiveness of silanization with selected functional silanes, from the point of view of improving the processing and operational properties of vulcanizates, in which CB is partially replaced with the finest fractions of fly ash. The silanization generally increased the interaction at the rubber–ash interface, while improving the degree of filler dispersion in the rubber mixture. The results of TGA and FTIR analyses confirmed the presence of silanes chemically bonded to the surface of fly ash particles. SEM tests and determination of the bound rubber (BdR) content show that the introduction of the silanes to the mixture increases the degree of ash dispersion (DI) and the Payne effect, which is the greatest when mercaptosilane was used for modification. The highest increase in torque, which was recorded in the case of rubber mixtures containing sulfur silanes and silane with epoxy groups, may be due to their participation in the vulcanization process, which is confirmed by the results of vulcametric studies. The lowest values of mechanical strength, elongation at break, and the highest hardness of vulcanizates obtained in this case may be the result of the over-crosslinking of the rubber. The addition of sulfur-containing silanes significantly slowed down the vulcanization process, which is particularly visible (up to three times extension of the t_90_ parameter, compared to mixtures without silane) in the case of Si-266. The addition of silanes, except for Si-266 (with a polysulfide fragment), generally improved the abrasion resistance of vulcanizates. The Dynaslan MTMO silane (with mercapto groups) performs best in this respect. Proper selection of silane for the finest fraction of fly ash in the rubber mixtures tested allows for an increase in the mechanical strength of their vulcanizates from 9.1 to 17 MPa, elongation at break from 290 to 500%, hardness from 68 to 74 °ShA, and reduction in abrasion from 171 to 147 mm^3^.

## 1. Introduction

The use of fly ash as filler in rubber mixtures and plastics has already been reported in studies by other scientists. Fly ash used to be subjected to high-energy grinding and then admixed in low quantity as a secondary filler, replacing a small part (10%–20% by weight) of the primary filler (usually carbon black), for non-demanding applications [1,2]. Instead of grinding, the ash can also be sieved to obtain finer fractions [3,4]. The fine fractions are then activated using various kinds of treatment:

- mixing with oils containing 30%–50% by weight of the naphthenic fraction [5], fatty acids, and their salts [6];
- mixing with liquid rubbers, e.g., butadiene (BR) or hydroxylated butadiene rubbers [7,8];
- by application of coupling agents in the form of silanes [9,10,11], zirconates, aluminates, or esters of titanic acid [12,13]; or
- by application of organic amines (preferably from the group of ethanolamine and propanolamine) [14].

A hybrid modification is often used, involving the silanization of the ash surface, followed by its modification with liquid rubbers [15,16] or surface activation of the ash with fatty acids (usually stearic or octadecanoic acid) and subsequent coupling agent treatment [17].

Generally, modifying the surface of fillers is a common method of improving their activity level and reinforcing capacity in rubber mixtures [18]. On the other hand, not many scientific papers are devoted to the modification of fly ash and its use as a carbon black substitute. Interesting results have been achieved by Sitarz and coworkers, who proposed dividing the fly ash into fractions and their subsequent silanization with [3-(2-aminoethylamino)propyl]-trimethoxysilane [19]. The authors successfully used fly ash prepared in this way as a filler for conductive epoxy resins. As for the application in rubber mixtures, an interesting proposition is the use of fly ash modified with bis (3-triethoxysilyl) propyl tetrasulfide (TESPT) as a filler for butadiene rubber (BR) [20]. The same silane was used for the testing of the effect of filler-surface treatment on the properties of fly ash/natural rubber (NR) vulcanizates [21] and later tested in NR/styrene-butadiene rubber (SBR) blends [22]. Alkadasi et al., demonstrated the effect, of using a titanate coupling agent to modify the fly ash, on the mechanical properties of filled SBR [23]. Later on, the same authors confirmed the modification also works for the compatibilization of fly ash with chloroprene rubber (CR) [24]. The mechanical properties of vulcanizates filled with fly ash subjected to the modification are much higher compared to vulcanizates filled with native fly ash. In another work, TESPT-modified fly ash was incorporated into NR up to 80 phr using solution mixing, to produce composites. [25]. Garde and coworkers studied the effect of fly ash used in conjunction with TESPT coupling agent, applied as a reinforcing filler for polyisoprene (IR)-based vulcanizates [26]. The selection of a silane coupling agent was guided by the possibility of ensuring interactions between the functional groups of the silanes and the active sites present on the ash surface [27].

The first part of the work [28] concerned the use of various ash fractions as a filler for rubber mixtures. A different chemical and phase composition of FA fractions with different grain sizes was found and their influence on the properties of rubber mixtures with their participation was investigated.

The aim of the current study was to investigate:

1. silane chemisorption on the fractionated fly ash surface; and
2. influence of fly ash silanization on the properties of rubber mixtures and selected mechanical properties of their vulcanizates, containing modified fly ash, partially replacing technical carbon black.

In numerous publications available in the subject literature, the authors subjected either the native ash or FA after its mechanical grinding to silanization, with different results. In this sense, there are many scientific papers devoted to the modification of fly ash and its use as a carbon black substitute. The novelty of the proposed approach to FA valorization is based on the fact of the existence of significant differences in the chemical and phase composition between its fractions, confirmed in our previous work [28]. In this paper, we decided to check the efficiency of silanization in relation to selected ash fractions and its impact on the mechanical properties of rubber with their participation. The silanization of selected FA fractions was aimed at reducing the intramolecular interactions, while increasing the interactions at the rubber–filler interface [29]. We expected that the high silica content in the ash might be responsible for the agglomeration and poor FA dispersion in the rubber matrix.

## 2. Experimental Procedure

### 2.1. Materials

#### 2.1.1. Silanes Used for Fly Ash Modification

The selection of silane binders was guided by the possibility of a chemical reaction between the functional groups present on the surface of various ash fractions, determined on the basis of their phase analysis [28] as well as their ability to participate in rubber crosslinking.

Based on the analysis, the following silanes were selected for the modification of the fly ash surface, namely:

1. Vinyltrimethoxysilane-U-611 (Unisil Ltd., Tarnów, Poland) [30], a part methoxy group directly involved in chemical binding with silanol and hydroxy groups present on the surface of fly ash particles, also with vinyl groups likely to participate in rubber crosslinking.
2. 3-(glycidoxypropyl)trimethoxysilane-U-50 (Unisil Ltd., Tarnów, Poland) [31], a part methoxy group responsible for the filler binding, also expected reactivity of its epoxy group towards thiol modification of rubber macromolecules, which might be formed during a sulfur vulcanization on the rubber [32].
3. Vinyl-tris(2-methoxy-ethoxy)silane-LUVOMAXX VTMOEO DL50 C (Lehmann & Voss Co., Hamburg, Germany) [33], phlegmatized on the surface of calcium carbonate (50/50), especially recommended for compounds with fillers containing silanol-groups, e.g., silicic acids or silicates.
4. Mercaptopropyltrimethoxysilane-Dynaslan MTMO (Evonik Industries AG, Essen, Germany) [34], a bifunctional organosilane possessing a reactive organic mercapto and a hydrolyzable inorganic methoxysilyl group, used as an adhesion promoter to such inorganic substrates such as silica, quartz, sand, cristobalite, mica, kaolin, talc, other silicate fillers, and metals, as well as crosslinking agent.
5. Bis(triethoxysilylpropyl)polysulfide-Si-266 (Evonik Industries AG, Essen, Germany) [35], reacting with silanol groups of white fillers during mixing and with rubber macromolecules during vulcanization, forming covalent chemical bonds.

For ease of reading, the structural formulas of the above-mentioned silanes are provided in Table S1 of the Supplementary Materials.

#### 2.1.2. Rubber Compounds and Their Vulcanizates

Fly ash A (sample collected in 2017) was selected for further research, due to the higher content of its silicon compounds and theoretically better potential to be a reinforcing filler. This type of fly ash was characterized and investigated for rubber reinforcement in our previous study [28]. Fractionation of the fly ash was performed using a laboratory sieve shaker LPzE-3e (MULTISERW-Morek Ltd., Brzeźnica, Poland), operating with 0.250, 0.125, and 0.063 mm sieves. Experimental conditions applied: test time—3 min, amplitude—1.5 mm, vibration frequency—50 Hz. The process was carried out by sieving the ash until a constant mass was obtained on each sieve. The measurement error was calculated using the Student’s *t*-test for α = 0.005. Based on the results of the first part of the publication [28], it turns out that the best results were obtained for ashes of small and medium grain size: FA < 0.063 mm, 0.063 < FA < 0.125 mm, and 0.125 < FA < 0.250 mm. They have been selected to evaluate the effectiveness of various silane binders in rubber mixes.

Rubber mixes based on styrene-butadiene rubber (SBR) Ker 1500 (Synthos S.A., Oświęcim, Poland) containing CB (N-220) and the fractionated FA were prepared with a Brabender-Plasicorder 50 (Duisburg, Germany) laboratory internal mixer, under the following conditions:

1. Rotor speed—ca. 60 rpm;
2. Chamber fill factor—75%.

The sequence and share of components, time of mixing stages, and thermal conditions during mixing, are summarized in Table 1.

At each stage, the ram was lowered and raised in order to minimize dead zones during mixing. In the second step, a crosslinking unit (sulfur and accelerators) was added to the rubber mix on a WT300 FAMPA (Poland) laboratory two-roll mill and mixed for ca. 8 min.

Based on the results presented in the first part of the paper [28], it was decided to limit further studies on the effectiveness of various silane binders [30,31,33,34,35] to rubber mixes filled with 30 phr of the CB and 20 phr of FA, subjected or non-subjected to fractionation (FA-All) and silanized internally, i.e., during mixing.

Table 2 contains composition of rubber mixes filled with N 220 carbon black (CB) and fly ashes (FA) silanized in situ.

The amounts of silanes were converted in such a way that the number of moles of functional groups was the same in proportion to mass. For example:

- for U-50, we have 1 mole of functional groups per 236.3 g;
- for U-611, we have 1 mole of functional groups per 148.2 g.

So, we have a proportion:

5 [phr]—236.3 [g]

X [phr]—148.2 [g]

X = (5 × 148.2 [g])/236.3 [g]

X = 3.1 [phr]

It must be remembered that for silanes having more than one functional group, e.g., Si-266 (TESPD) decaying into two reactive molecules, the molecular mass will produce two moles of active groups, not just one.

Rubber samples were steel-moulded at 145 °C during optimum vulcanization time of t_90_, determined vulcametrically by a MDR 3000 oscillating disk rheometer (Alpha Technologies, Bellingham, WA, USA), according to PN-ISO 3417.

### 2.2. Methods

#### 2.2.1. Susceptibility of Fly Ash to Silanization

Surface functionalization of the fly ash by silanization was evaluated on its finest FA-63 fraction, subjected to an ex situ modification. The procedure of the silanization is as follows:

- 10 g of the ash was placed in 100 mL of toluene, with the addition of 0.2 mL of trifluoroacetic acid and a magnetic stirrer;
- it was heated to 105 °C and 2 mL of silane was added;
- the reaction mixture was kept at 105 °C for 5 h with constant stirring;
- the obtained modified ash was filtered, rinsed, and dried at 110 °C for 2 h.

The effect of fly ash treatment by vinyl silanes (U-611 and LUVOMAXX VTMOEO DL50 C) was analyzed by Fourier transform infrared (FTIR) spectroscopy using a Nicolet IS 50 spectrometer (Thermo Fisher Scientific Inc., Bartlesville, OK, USA) with an EASIDIFF DRIFT (diffuse reflectance infrared Fourier transform spectrometry) cell (PIKE Technologies). MCT (Mercury-Cadmium-Telluride) detector working in the spectral range of 4000–650 cm^−1^ with 8 cm^−1^ optical resolution and accumulating 32 scans was used to register the spectra of each powder tested. DRIFT spectra of functionalized FA powders were analyzed looking for the characteristic absorption bands of all the silanes studied [36].

The mixtures of 600 mg of dried potassium bromide (KBr) and 100 mg of ash were made and carefully ground in an agate mortar. The tested samples were mixed in precisely the same proportion. All weights were made on an analytical balance with an accuracy of 0.1 mg. An infrared spectrum of fly ash (FA) activated with a mixture of trifluoroacetic acid (TFA) and toluene was used as a background when analyzing the FA spectra after silanization. All spectra of the tested samples were recorded using a reference spectrum containing unmodified ash. In this way, the signals in the acquisited spectra came from substances that were introduced on the surface of the samples during their modification, with the simultaneous compensation of most of the signals coming from the tested ash substrate, originating mainly from alkaline metal oxides [28].

The quantity of silane attached to the fly ash was determined by means of Thermogravimetric Analysis (TGA) using a TG 209 device (Netzsch, Selb, Germany), operating under an air atmosphere with a heating rate of 20 °C/min from room temperature to 800 °C. The percentage weight loss for each silanization case was calculated as the result of the value difference in the range of 280–330 °C. The data used for the calculations were obtained as a result of the analysis performed with the software for the TG 209 device. The range of changes under the influence of silanization was determined by referring the FTIR and TGA spectra of the modified fly ash to its spectra from before the modification.

#### 2.2.2. Morphology of Rubber Vulcanizates

Morphological analysis of the rubber vulcanizates was carried out with the use of a scanning electron microscope (SEM) Nova Nano Sem 200f (FEI, UK) cooperating with the energy dispersive X-ray spectrometer (EDS), which enables the analysis of the chemical composition. Observations of the microstructure were carried out on samples’ cross-sections covered with a thin layer of carbon, using a reflected electron (BSE) detector with an accelerating voltage of 18 kV, operating under high vacuum conditions, or a secondary electron detector (LVD) with an accelerating voltage of 10–18 kV, operating under low vacuum conditions (60 Pa).

Additionally, the filler dispersion in rubber vulcanizates was determined using a DisperTester 3000 instrument (Montech, Columbia City, IN, USA), according to ISO 11345:2006. Filler dispersion (D), denoting the degree of filler dispersion in the vulcanizate, is described by the formula:

D = (1 − N_a_/N_tot_) × 100%

where:

N_a_—total number of pixels containing agglomerates (white areas);

N_tot_—total number of pixels in the image.

Data processing was carried out with the help of MonDispersion software, delivered with the instrument. Magnification applied was ×1000, with all the images referring to an area of 250 × 150 µm.

#### 2.2.3. Specific Surface Area of Fly Ash Particles

The specific surface area (SSA) of the fly ash and its fractions were determined with an ASAP 2010 (Micromeritics, Norcross, GA, USA) instrument, applying the Brunauer–Emmet–Teller (BET) equation. The samples were degassed at 160 °C for 24 h, and the obtained vacuum value was 2 µTr. The measurement was carried out at the temperature of liquid nitrogen. Specific Surface Area (SSA) was determined utilizing a 5-point BET procedure, according to ASTM D3037 and ASTM D4820 standards. The measurement error was calculated using the Student’s *t*-test for α = 0.005.

#### 2.2.4. Bound Rubber Content (BdR)

Bound rubber content of the rubber compounds was determined by extracting the unbound components such as free rubber and other ingredients with toluene at room temperature for 6 days and afterwards with n-hexane at room temperature for 1 day. Then, the samples were dried at room temperature for 2 days. Weights of the samples before and after the extraction were measured and the bound rubber contents were calculated by equation:

$$BdR\;\left(\%\right)=100\times \{W_{fg}-W_{t}\left[\frac{m_{f}}{\left(m_{f}+m_{r}\right)}\right]/\left\{W_{t}\left[m_{r}/\left(m_{f}+m_{r}\right)\right]\right\}\}$$

where *BdR* is the bound rubber content, *W_fg_* is the weight of filler and gel, *W_t_* is the weight of the sample, *m_f_* is the fraction of the filler in the compound, and *m_r_* is the fraction of the rubber in the compound [37]. Extraction procedure of rubber component (unbound rubber) from the compound was as follows: (1) unbound rubber chains were extracted from the compound with toluene at room temperature, (2) ethanol was gradually added to coagulate the polymer component, and (3) the coagulated rubber was washed with ethanol and dried in a vacuum oven.

#### 2.2.5. Payne Effect

Payne effect of the unvulcanized rubber compounds was examined using a MonTech D-RPA 3000 (Germany), operating with the strain sweep from 0.56% to 100% at 100 °C. The Payne effect of the rubber samples was determined as the difference between storage moduli at the highest (100%) and the lowest (0.56%) deformations.

#### 2.2.6. Mechanical Properties of Rubber Vulcanizates

The rubber vulcanizates containing the fly ashes or their fractions, subjected to static elongation, were examined with a 1435 universal mechanical testing machine operating with an optical extensometer (Zwick/Roell, Ulm, Germany), according to PN-ISO 37. Then, 2.0 ± 0.2 mm thick and 25 mm long (measuring distance) dumbbell specimens were elongated with speed of 500 mm/min. Next, 6 samples per material were tested, and the experimental values averaged. Stress at elongation of 100%, 200%, and 300%—SE 100, SE 200, and SE 300, respectively, tensile strength—TS, as well as the elongation at break—Eb—were determined. Hardness of the rubber vulcanizates was determined with a Zwick Shore A durometer 3130 (Zwick/Roell, Ulm, Germany), according to PN-EN ISO 868. The measurement error was each case calculated using the Student’s *t*-test for α = 0.005.

#### 2.2.7. Abrasion Resistance of Rubber Vulcanizates

Abrasion resistance of rubber vulcanizates was determined with a Schopper-Scholbach instrument (VEB Thuringer Industriewerk Raunstein, Berlin, Germany, according PN-ISO 4649:2007, met. A).

## 3. Results and Discussion

### 3.1. Susceptibility of Fly Ash to Silanization

**Figure 1:**
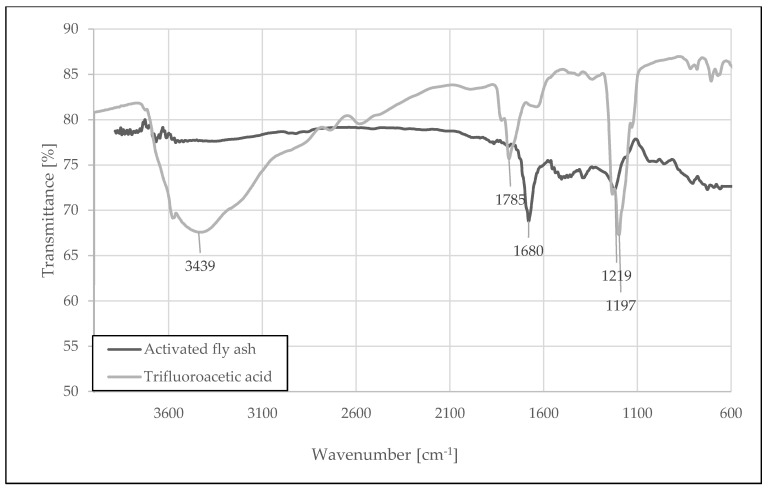
FT-IR spectra of the activated fly ash (black) compared to pure trifluoroacetic acid (gray).

As we can see in Figure 1, the process of ash activation slightly changes the chemical composition of its surface. Apart from adsorption maxima at 1200 cm^−1^ (1199 cm^−1^ for trifluoracetic acid and 1224 cm^−1^ for modified fly ash), characteristic for the -CF3 group, the shift of the absorption band characteristic for the carbonyl group in CF3-CO-O- from 1785 cm^−1^ to 1680 cm^−1^ corresponds to the conversion of free trifluoroacetic acid into its salt, which is also visible. This is additionally confirmed by the disappearance of the wide band with the maximum at 3423 cm^−1^, characteristic for the -OH groups present in carboxylic acids [38].

Based on this observation, it can be concluded that the surface of alkaline metal oxides present in the ash (calcium, sodium, iron, and other oxides, the presence of which is in the ash [28]) is covered with a thin coating of their salts with trifluoroacetic acid. The band characteristic for the salt of trifluoroacetic acid (1680 cm^−1^) with varying intensity is present in the spectra of all the ashes tested. Other absorption bands at 1673, 1100, and 1047 cm^−1^, originated from the residues of trifluoroacetic acid (TFA), can still remain on the surface of FA particles after ex situ modification [38,39]. TFA is hard to remove from the surface of mineral particles subjected to an acid treatment.

**Figure 2:**
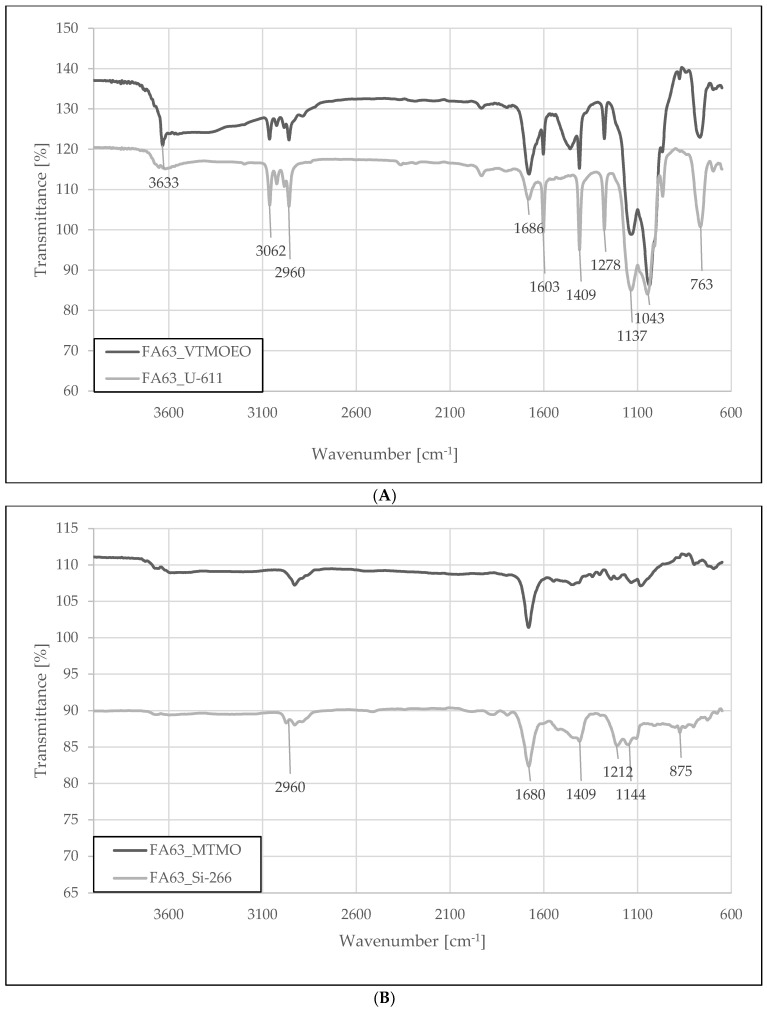
Fourier transform infrared (FTIR) spectroscopy analysis of FA-63 fraction modified by vinyl (**A**) and sulfur-containing (**B**) silanes.

Silanization of fly ash was confirmed on the example of its FA-63 fraction. The spectra of the FA samples treated with vinyl silanes, U-611 or LUVAMAX VTMOEO silane (Figure 2A), show the adsorption bands characteristic for vinyl groups attached to the silica network. This is evidenced by the presence of bands corresponding to the stretching (3062 and 3025 cm^−1^) and deformation (1602 and 1410 cm^−1^) vibrations of the vinyl group. A broad band with a maximum at 1130 cm^−1^ corresponds to the vibrations of the Si-O-Si groups in the silica lattice. However, the presence of the bands characteristic for the methoxy group (2984, 2954, and 1277 cm^−1^) indicates incomplete hydrolysis of the modifiers used for these samples.

The effect is much less visible for epoxide, polysulfidic, and mercapto silanes—Figure 2B.

**Figure 3:**
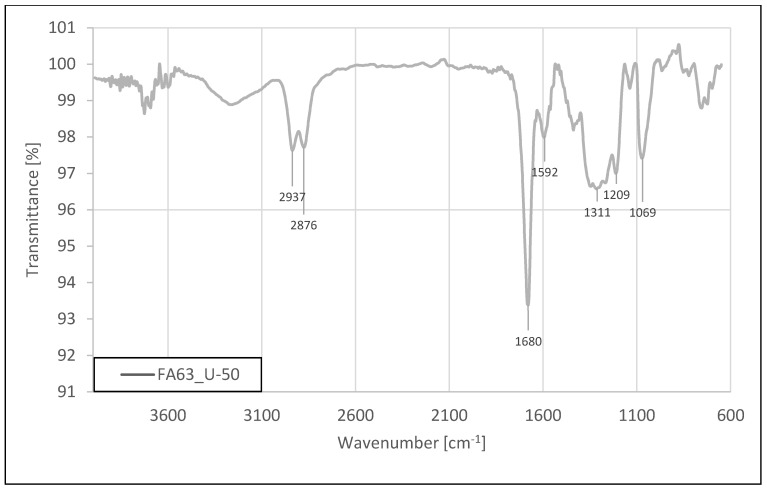
Fourier transform infrared (FTIR) spectroscopy analysis of FA-63 fraction modified by 3-glycidoxypropyltriethoxysilane (U-50).

The infrared spectrum of U-50 (3-glycidoxypropyltriethoxysilane) functionalized ash sample is shown in Figure 3.

Apart from the dominant absorption band characteristic for the salt of trifluoroacetic acid with the maximum at 1680 cm^−1^, there are also visible bands from the modifier with maxima at 2937 and 2876 cm^−1^, characteristic for the -CH_2_- groups, proving the presence of a small amount of the silane on the surface of ash particles. Probably, the epoxy group from the modifier molecule could partially react with the surface because its characteristic band, usually present at around 3075–3030 cm^−1^, is not visible. The presence of the 1074 cm^−1^ band mainly indicates the presence of Si-O-Si bonds from the silica, but it may also contain a signal from C-O groups. The results obtained confirmed that functionalization took place; however, the amount of silane on the ash surface is much smaller than in the case of the modifiers containing vinyl groups.

**Figure 4:**
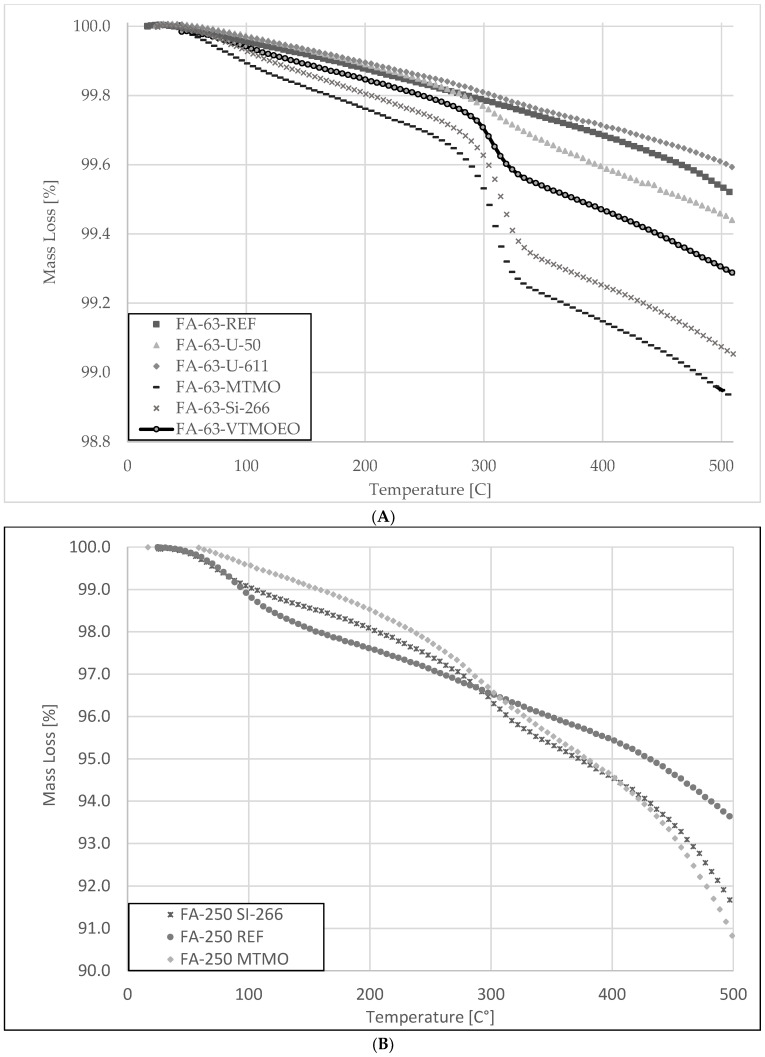
Thermogravimetric analysis (TGA) of the unmodified and silane modified fly ash: (**A**) fraction < 0.063 mm; (**B**) 0.125 mm < fraction < 0.250 mm.

The amount of silane that was attached to the FA-63 fraction was quantified by TGA—Figure 4.

The quantity of modifying agents at the surface of the FA fraction (A) particles was approximately 0.376% for MTMO silane, 0.253% for Si-266 silane, 0.199% for VTMOEO silane, 0.109% for U-50 silane, and 0.056% for U-611 silane. The quantity of modifying agents at the surface of the FA fraction (B) particles was approximately 1.188% for Si-266 silane and 1.170% for MTMO silane.

After separating the fly ash into fractions and carrying out silanization, some dependencies can be seen, namely:

- The bigger the ash grains are, the higher the weight loss during heating. Bigger grains have more developed structures and have the potential to contain liquid compounds, evaporating upon heating.
- The loss of mass begins below 100 °C—which indicates the presence of volatile fractions. It is very likely that some of them are water because this fraction decreases for the MTMO sample, i.e., as if it was consumed in the silanization process. In addition, bigger grains contain more carbon compounds, which are burnt during the measurement, as the process is carried out in the air. Water is one of the main products of carbon combustion, so it seems likely that its large proportion will adsorb on the porous surface of the biggest FA particles of the highest SSA.
- Better results were obtained for the finer ash fractions, which contain more mineral compounds (mainly silica), capable of chemically reacting with silanes. Bigger ash fractions, containing more carbon compounds, are not able to chemically react with silanes, but it can be seen that at least some of them are deposited on the present mineral phases (weight loss at 300 °C).

### 3.2. Morphology of Rubber Vulcanizates

**Figure 5:**
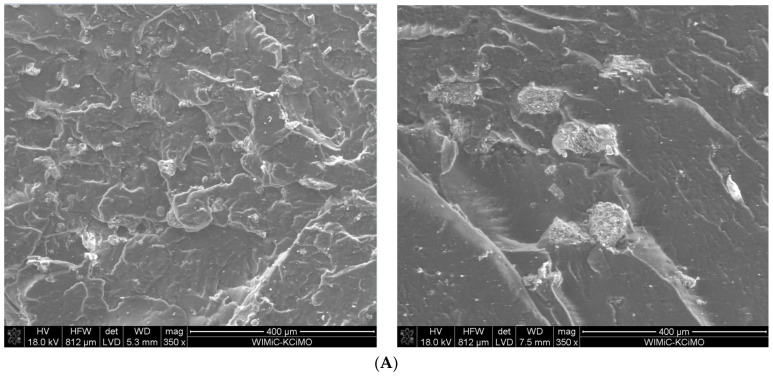

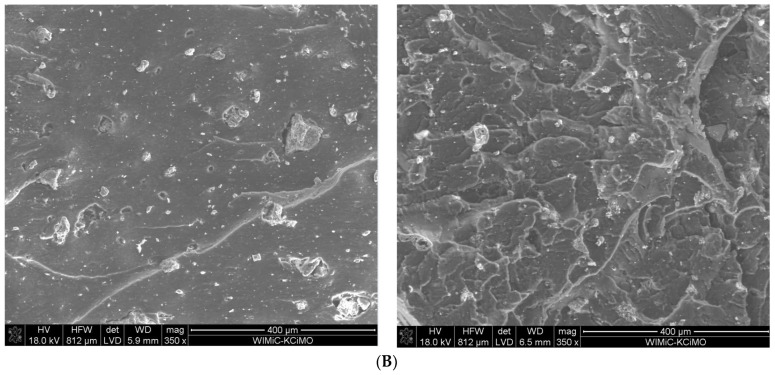
SEM pictures of the vulcanizates containing 30 phr of N 220 and 20 phr of fractionated fly ash (FA) with the addition of U-50 silane (left) or Si-266 silane (right) for: (**A**) FA-63 and (**B**) FA-125.

The addition of each of the silanes improves the dispersion of fillers in the rubber matrix, which was illustrated by selected examples of tests using the SEM method—Figure 5.

The degree of dispersion of fillers containing carbon black and various fractions of fly ash treated with different silanes is summarized in Table 3.

Comparing the degree of filler dispersion for mixtures with the finest ash (FA-63), the effects caused by adding different silanes are very similar to each other. As for the next higher fraction (FA-125), in most cases there is also an improvement in dispersion due to the addition of silanes. The exceptions are the VTMOEO and MTMO silanes. Again, the best results were obtained with the U-50 and Si-266 silanes, where the increase in the degree of dispersion is much higher than with the smaller fraction. As expected, the degree of dispersion increases with increasing grain size of the ash fraction, which is in line with the generally accepted principles in elastomer technology.

### 3.3. Bound Rubber Content (BdR)

Silanization of virgin fly ash makes its specific surface area (BET) significantly decreased and equal, no matter the kind of silane applied—Table 4. This might be the effect of occupying the specific active sides on the filler surface by the silane molecules and the TFA residues. Their chemisorption on the fillers reduces the accessibility of the surface for the N_2_ molecules during the BET analysis.

**Figure 6:**
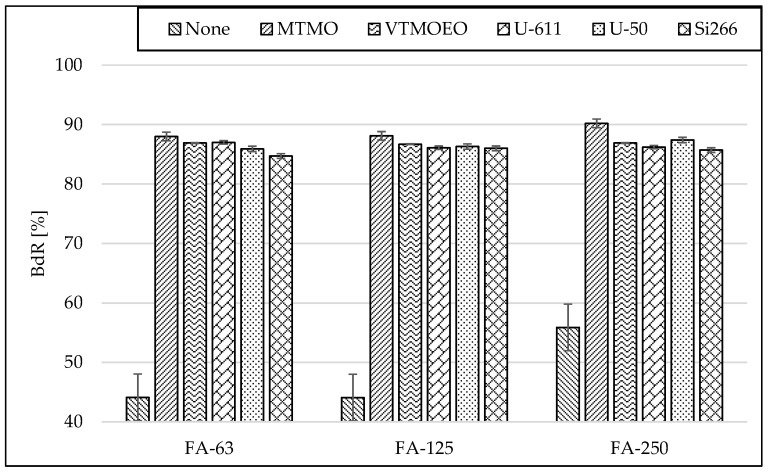
Influence of silanization on the Bound Rubber content (BdR) in rubber vulcanizates containing 30 phr of N 220 and 20 phr of fractionated fly ash.

Despite the significantly lower SSA value of silanized FA in comparison to the unmodified particles, the modifications make rubber–filler interactions significantly increase—Figure 6.

It can be clearly seen that the addition of each of the silanes significantly increases the Bound Rubber content (BdR). This is in line with the literature data [40]. However, there is no significant difference in the BdR value between the individual fractions of modified ash, as there was even before the modification. The significant increase in BdR content might be a result of the oligomerization of the silanes. All the used silanes contain three hydrolysable groups, which are responsible for bonding to fillers’ surfaces. However, simultaneously a condensation reaction between the groups takes place [41]. As a result, oligomeric structures are formed, which affect rubber properties. Such a phenomenon was hypothesized to be responsible for rubber reinforcement [42] and changes in the kinetics of rubber vulcanization [43]. In this case, the silane oligomer molecules can entangle with the rubber matrix and improve the interfacial adhesion between the fly ash and the rubber. As a result, this provides a high increase in the BdR content, regardless of the type of the silane functional group. Despite the similar effects obtained, no matter the kind of silane used, slightly higher BdR values were obtained for the rubber mixtures modified with silanes containing mercapto groups, which is likely to be the result of their contribution to vulcanization. It was confirmed that the different sulfur species of the silanes interact with polymer, which has a significant influence on the processability, and filler dispersion state, eventually resulting in the mechanical properties of the final rubber compound [44].

### 3.4. Payne Effect

**Figure 7:**
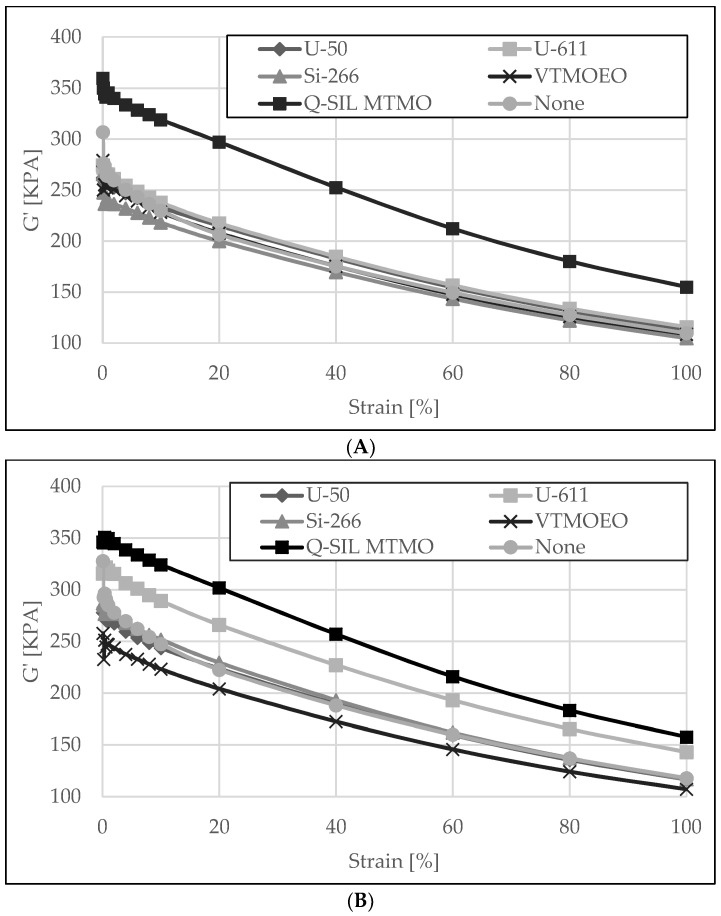

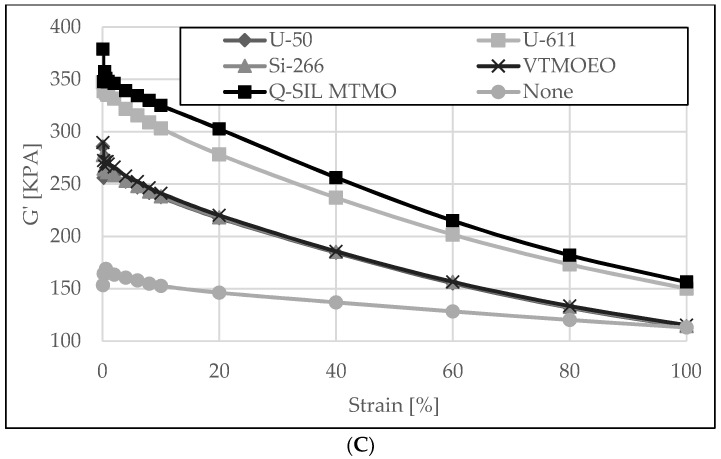
Payne effect for the rubber compounds filled with 30 phr of N-220 and 20 phr of unmodified or silanized: (**A**) FA-63; (**B**) FA-125; (**C**) FA-250.

The influence of the admixing of different silanes to SBR filled with 30 phr of N-220 and 20 phr of fly ash on the Payne effect of the compounds was determined for various FA fractions, which is illustrated in Figure 7 and compared in Table 5.

The observed changes to ΔG′ stay in agreement with the previous studies on dispersion, and bound rubber content. The efficiency of silanization seems not to be related to increasing polymer–filler interactions, which seem to be similar (to BdR), but depend rather on filler microdispersion, related to a different Payne effect.

### 3.5. Kinetics of Vulcanization of Rubber Compounds

**Figure 8:**
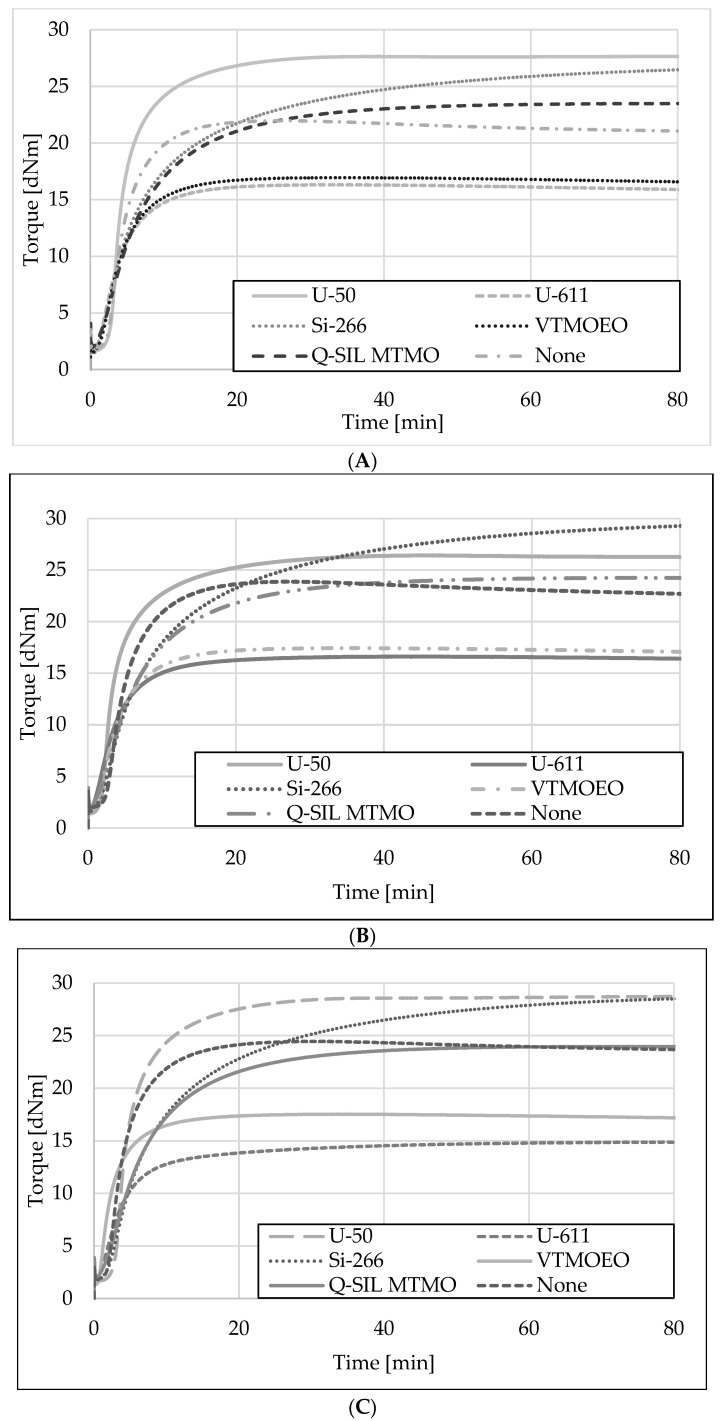
Vulcanization kinetics of the rubber compounds filled with 30 phr of N-220 and 20 phr of unmodified or silanized: (**A**) FA-63; (**B**) FA-125; (**C**) FA-250.

The vulcanization kinetics of the rubber mixtures filled with 30 phr of N 220 and 20 phr of fly ash fractions and modified by the addition of different silanes are illustrated in Figure 8, and the parameters of their vulcanization are presented in Table 6.

**Figure 9:**
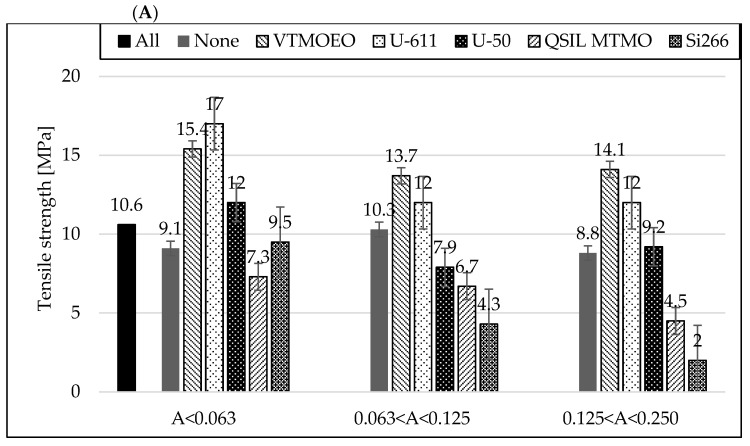

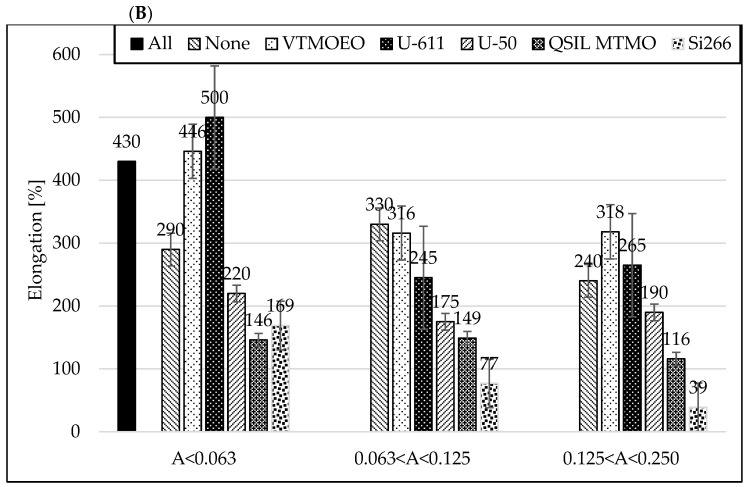
Influence of the silanization of fly ash with various silanes on mechanical properties: TS (**A**) and elongation at break (**B**) of the rubber vulcanizates filled with CB and different FA fractions.

Despite slight changes in the individual values described in Table 6, regardless of the selected ash fraction, the vulcanization kinetics look similar. It is clearly visible that the mixtures modified with sulfur-containing silanes have the highest values of the optimum vulcanization (almost three times higher than other mixtures). Mixtures of silanes with vinyl groups have the lowest values of maximum torque, which translates directly into the best elasticity of vulcanizates. It is likely that the oligomers formed due to the vinyl-silanes (U-611 and VTMOEO) condensation, on one hand, act as plasticizers because of their less polar nature in comparison to the other silanes but, on the other hand, take part in the vulcanization forming an alternative in-rubber structure that reinforces and contributes to the elasticity of the system. Such reduction in torque during the kinetics of vulcanization tests was previously observed in the literature with a similar effect on the elongation at break [45]. Differently, the mixtures with Si-266 silane containing polysulfide functionality in its structure have the highest M_max_ value, which is likely to affect the vulcanization, giving them too high stiffness, which is responsible for the worst mechanical parameters. The FTIR spectra show that silanes with vinyl groups adsorb the best on the ash surface, while those containing epoxy or sulfur groups (mercapto group—MTMO or polysulphides—Si 266) are worse. A higher increase in the vulcametric torque recorded in the case of rubber mixtures containing silanes with epoxy or sulfur groups may be caused by the participation of the latter in sulfur vulcanization [46,47], which is confirmed by the results of vulcametric tests, the lowest values of mechanical strength, and the lowest elongation of vulcanizates at break—Figure 9. Analyzing the results from Table 6, it can be seen that the vulcametric torques for native ashes of different fractions and their equivalents with Dynaslan MTMO silane show a similar value, while the addition of Si-266 silane significantly increases the value of this parameter. Moreover, the addition of silanes significantly slows down the vulcanization process. For mixtures with the addition of Dynaslan MTMO, the t_90_ parameter is two times longer, while for Si-266 it is even three times longer, compared to mixtures without silanes. Extending the vulcanization time may result in the “maturing of network” effect, modifying the structure of sulfidic crosslinks towards increasing the number of short: mono- and disulfidic crosslinks at the expense of polysulfidic ones [48]. Such a modification may contribute to the observed increase in the stiffness of the vulcanizates.

### 3.6. Mechanical Properties of Rubber Vulcanizates

**Figure 10:**
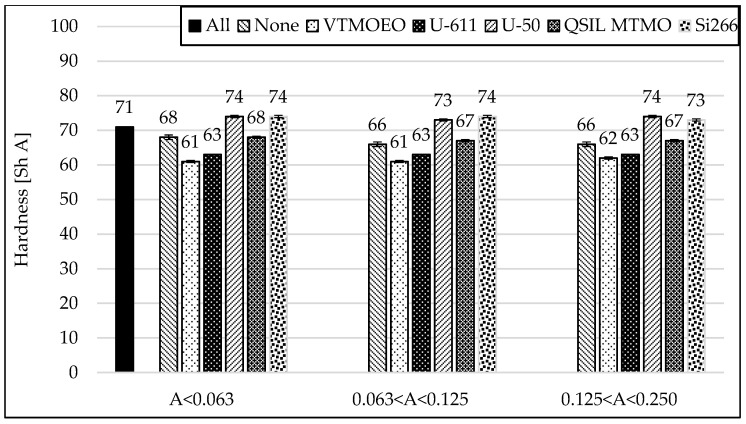
Influence of the silanization of fly ash with various silanes on hardness of the rubber vulcanizates filled with CB and different FA fractions.

When analyzing Figure 9 and Figure 10 and Table 7, it is clearly seen that the best strengthening effects were obtained for the mixtures containing the finest ash. In addition, a positive aspect is the maintenance of a very good elongation of the vulcanizates at break. For other, bigger fractions, when using different silanes, there is a clear tendency indicating that the larger the size of the ash grains is, the lower the elongation at break values and the lower the tensile strength of the vulcanizates. 

Despite the higher surface energy and larger specific surface area [28], the biggest FA grains can be the places of crack propagation, hence, probably with lower values of tensile strength and elongation at break of the vulcanizates. However, if the phenomenon of grains agglomeration in the smallest FA fraction is avoided, it will bring the best strength results. The highest values of strength and elongation at break, with slightly lower hardness, were obtained for the vulcanizates containing coupling agents with vinyl groups (U-611 and VTMOEO). The worst results were obtained for the vulcanizates with silanes containing sulfur in their structures (Si-266 and Dynaslan MTMO), which turned out to be the stiffest and quickly cracked under elongation.

Si-266 silane has two types of chemically reactive groups: ethoxy and polysulfide. The ethoxy groups react with the silanol groups on the fly ash surface during blending by hydrolysis, while the sulfur of the polysulfide group reacts with a double bond in the rubber macromolecules in the presence of accelerators in the vulcanization step. Thus, a covalent network is formed between the rubber macromolecules and the FA containing the silane compatibilizer. As for the Dynaslan MTMO silane, the methoxy group can hydrolyze and condense with the hydroxyl groups present on the fly ash surface. Then, during vulcanization, the carbocations from the SBR molecules form C-S bonds by reaction with the thiol groups formed in the silane. In this way, the silane also connects the fly ash to the rubber macromolecules by covalent bonds.

The difference in the silanization effects between the abovementioned coupling agents was manifested in the fact that by increasing the amount of polysulfide silane introduced into the mixture, the mechanical strength of vulcanizates increases slightly so that after exceeding 13 phr, it begins to decrease significantly, whereas in the case of silane with the mercapto group, the higher the amount of silane is added to the mixture, the mechanical strength and elongation of the vulcanizates at break decrease and their hardness increases.

Probably, due to the negligible content of metals with variable valence in the composition of the fly ash [28], they do not affect the aging of vulcanizates, if it is evidenced by the lack of changes in their hardness as a result of thermal aging, as demonstrated by the data collected in Table S2 of the Supplementary Materials.

### 3.7. Abrasion Resistance of Rubber Vulcanizates

**Figure 11:**
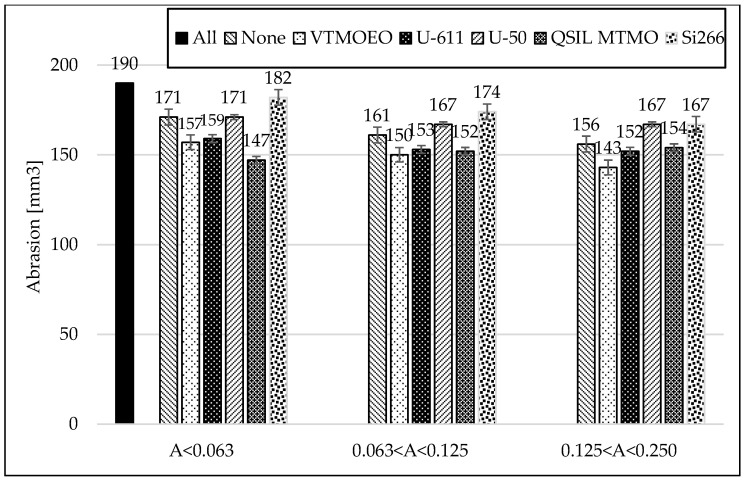
Influence on the silanization of fly ash with various silanes on abrasion of the rubber vulcanizates filled with CB and different FA fractions.

The results of the abrasion test clearly show that, firstly, the division of ash into fractions improves this parameter [28]. In addition, the modification with most silanes even allows to improve the obtained results—Figure 11.

Only silanization with the Si-266 coupling agent (containing polysulfide functionality) for each fraction increases the abrasion compared to the vulcanizates without the addition of silane. Interestingly, the second sulfur silane with mercapto groups (Dynaslan MTMO) perform very well, as do vinyl silane coupling agents (U-611 and VTMOEO). From the point of view of some technical applications, this is a very big advantage and worth noticing.

## 4. Conclusions

Ex situ silanization studies show that all of the coupling agents tested are chemically bonded to the fly ash surface. This is indicated by both the decrease in the specific surface area (BET) value of the ash and the results of the TGA and FT-IR analyses. The sulfur silanes tested adsorb on the FA surface in higher amounts than the silane with the epoxy group or the vinyl silanes (TGA).

In situ silanization causes a significant increase in the content of bound rubber, regardless of the size of the FA fraction and the type of functional silane used, and an improvement in the degree of filler dispersion in the rubber matrix. The most effective in the latter case is the addition of mercaptosilane, which is confirmed by the Payne effect analysis results for the tested vulcanizates containing carbon black, various FA fractions, and silanes differing in chemical functionality. It is highly likely that apart from a classic filler-polymer coupling via silane bridges, silane oligomerization also takes place that can form an alternative in-rubber structure, significantly affecting the mixes’ properties.

The possible participation of silanes with epoxy and sulfur groups in the vulcanization process of rubber manifests itself by a reduction in the mechanical strength and elongation of the vulcanizates at break. In the case of the sulfur-functionalized silanes, the “maturing of network” effect [48] can contribute to the observed increase in the stiffness of the vulcanizates.

The addition of vinyl silanes and mercaptosilane is also advantageous from the point of view of reducing the abrasiveness of the vulcanizates containing carbon black and fly ash.

## Figures and Tables

**Table 1:** Mixing process for the rubber compounds containing fly ash silanized in situ.

Sequence of the Ingredients Addition	Time from Mixing Start [min]/Temperature [°C]
Adding rubber (SBR; Ker 1500)	0/25
Adding CB, stearic acid, and ZnO	1.5/90
Adding fly ash and ½ silane	3/100
Adding ½ silane	4/135
End of mixing	8–9/max. 140

**Table 2:** Formulation of the rubber mixes filled with CB, fly ash, and silanes [phr].

Rubber Compound Components [phr]	CB-FA-All	CB-FA-250	CB-FA-250-U-50	CB-FA-250-U-611	CB-FA-250-Si-266	CB-FA-250-VTMOEO	CB-FA-250-MTMO	CB-FA-125	CB-FA-125-U-50	CB-FA-125-U-611	CB-FA-125-Si-266	CB-FA-125-VTMOEO	CB-FA-125-MTMO	CB-FA-63	CB-FA-63-U-50	CB-FA-63-U-611	CB-FA-63-Si-266	CB-FA-63-VTMOEO	CB-FA-63-MTMO
SBR, Ker 1500	100																		
Stearic acid	3																		
ZnO	5																		
Carbon black, N 220	30																		
FA-All	20	-	-	-	-	-	-	-	-	-	-	-	-	-	-	-	-	-	-
0.125 < FA < 0.250	-	20	20	20	20	20	20	-	-	-	-	-	-	-	-	-	-	-	-
0.125 > FA > 0.063	-	-	-	-	-	-	-	20	20	20	20	20	20	-	-	-	-	-	-
FA < 0.063	-	-	-	-	-	-	-	-	-	-	-	-	-	20	20	20	20	20	20
Silane U-50	-	-	5	-	-	-	-	-	5	-	-	-	-	-	5	-	-	-	-
Silane U-611	-	-	-	3.1	-	-	-	-	-	3.1	-	-	-	-	-	3.1	-	-	-
Silane Si-266	-	-	-	-	5.7	-	-	-	-	-	5.7	-	-	-	-	-	5.7	-	-
Silane VTMOEO	-	-	-	-	-	5.9	-	-	-	-	-	5.9	-	-	-	-	-	5.9	-
Silane QSIL MTMO	-	-	-	-	-	-	4.1	-	-	-	-	-	4.1	-	-	-	-	-	4.1
Sulfur	2																		
N-cykloheksylo-2-benzothiazyl sulfenamide, CBS	1																		
Tetramethylthiu-ram disulfide, TMTD	1																		

**Table 3:** Influence of silanization on the filler dispersion of rubber vulcanizates containing 30 phr of N 220 and 20 phr of the fractionated fly ash.

Filler System + Silane	Dispersion, D [%]
CB+FA-63	30.8
CB+FA-125	45.5
CB+FA-250	60.0
CB+FA-63-U-50	43.3
CB+FA-125-U-50	84.7
CB+FA-250-U-50	59.8
CB+FA-63-U-611	34.0
CB+FA-125-U-611	50.9
CB+FA-250-U-611	64.6
CB+FA-63-Si-266	39.3
CB+FA-125-Si-266	58.6
FA-250-Si-266	52.0
CB+FA-63-VTMOEO	34.3
CB+FA-125-VTMOEO	45.0
CB+FA-250-VTMOEO	52.4
CB+FA-63-MTMO	34.0
CB+FA-125-MTMO	42.5
CB+FA-250-MTMO	61.4

**Table 4:** BET specific surface area (SSA) of unmodified and silanized virgin fly ash.

FA Treatment	BET [m^2^/g]
Unmodified	18.2 ± 0.03
U-611	6.36 ± 0.03
U-50	5.69 ± 0.01
VTMOEO	5.56 ± 0.01

**Table 5:** Payne effect for the rubber compounds filled with 30 phr of N-220 and 20 phr of unmodified or silanized FA fractions.

	Without Silane	U-50	U-611	VTMOEO	Q-SIL MTMO	Si-266
**FA-63**						
**G′ at 100%**	109.6	113.3	115.7	108.6	154.9	104.8
**G′_max_-G′_min_**	166.5	154.8	156.4	146.7	195.1	142.7
**FA-125**						
**G′ at 100%**	118.0	116.7	143.1	107.2	157.7	116.8
**G′_max_-G′_min_**	174.7	161.2	176.5	144.0	193.1	168.1
**FA-250**						
**G′ at 100%**	112.9	113.4	150.0	115.1	156.4	114.3
**G′_max_-G′_min_**	51.4	162.2	188.7	157.2	194.5	150.3

**Table 6:** Vulcanization parameters of rubber mixtures filled with 30 phr of N-220 and 20 phr of different sizes of FA and treated/untreated by different silanes.

Parameter Sample	t_90_ [min]	t_02_ [min]	M_min_ [dNm]	M_max_ [dNm]	ΔM [dNm]
CB + FA-63	10.2	2.5	1.7	22.0	20.3
CB + FA-125	11.4	2.6	1.8	23.9	22.1
CB + FA-250	10.6	2.1	1.7	24.4	22.7
CB + FA-63-U-50	12.0	2.7	1.6	27.6	26.0
CB + FA-125-U-50	13.3	1.9	1.4	26.4	25.0
CB + FA-250-U-50	13.5	2.8	1.6	28.8	27.2
CB + FA-63-U-611	10.3	1.5	1.5	16.3	14.8
CB + FA-125-U-611	10.3	1.3	1.7	16.6	14.9
CB + FA-250-U-611	15.8	1.5	1.8	14.9	13.1
CB + FA-63-Si-266	33.7	1.8	1.4	26.6	25.2
CB + FA-125-Si-266	36.9	2.0	1.6	29.5	27.9
FA-250-Si-266	35.4	2.3	1.6	28.7	27.1
CB + FA-63-VTMOEO	10.6	1.8	1.5	16.9	15.4
CB + FA-125-VTMOEO	10.4	1.6	1.5	17.4	15.9
CB + FA-250-VTMOEO	7.9	1.0	1.4	17.5	16.1
CB + FA-63-MTMO	21.4	1.8	2.0	23.5	21.5
CB + FA-125-MTMO	21.1	1.8	2.0	24.3	22.3
CB + FA-250-MTMO	20.8	1.9	2.0	24.0	22.0

t_90_—optimum vulcanization time; t_02_—scorch time; M_min_—minimum torque; M_max_—maximum torque; ΔM—increase in vulcametric torque.

**Table 7:** Influence of the silanization of fly ash with various silanes on stress at elongation of 100%, 200%, and 300% of the rubber vulcanizates filled with CB and different FA fractions.

	Parameter	SE 100 [MPa]	SE 200 [MPa]	SE 300 [MPa]
Sample				
CB + FA		4.4	6.2	9.5
CB + FA-63		4.0	6.6	-
CB + FA-125		4.3	6.3	9.8
CB + FA-250		3.8	5.9	-
CB + FA-63-U-50		4.2	10.2	-
CB + FA-125-U-50		4.0	-	-
CB + FA-250-U-50		4.2	-	-
CB + FA-63-U-611		1.4	4.2	8.4
CB + FA-125-U-611		3.4	8.8	-
CB + FA-250-U-611		3.0	7.2	-
CB + FA-63-Si-266		4.6	-	-
CB + FA-125-Si-266		-	-	-
FA-250-Si-266		-	-	-
CB + FA-63-VTMOEO		1.8	3.4	7.4
CB + FA-125-VTMOEO		2.4	7.0	12.9
CB + FA-250-VTMOEO		2.4	7.1	13.0
CB + FA-63-MTMO		4.8	-	-
CB + FA-125-MTMO		4.0	-	-
CB + FA-250-MTMO		3.8	-	-

## Data Availability

The data presented in this study are available on request from the corresponding author. The data are not publicly available due to [part of industrial project].

## Supplementary Materials

The following supporting information can be downloaded at: https://www.mdpi.com/article/10.3390/ma15175979/s1. Table S1. Structural formulas of the silanes used for the modification of FA. Table S2. Influence of thermal aging on hardness (H) of the rubber vulcanizates.
