# Supplementary material for: Fly Ash from Lignite Combustion as a Filler for Rubber Mixes—Part II: Chemical Valorisation of Fly Ash
Source: Materials (Basel). 2022 Aug 29;15(17):5979. doi: 10.3390/ma15175979 (PMC9456590; doi:10.3390/ma15175979)
Supplement: Supplementary file 1 [file materials-15-05979-s001.zip › materials-1862047-supplementary.pdf]

Table S1. Structural formulas of the silanes used for the modification of FA.

Silane	Structural formula
Vinyltrimethoxysilane<br>- U-611	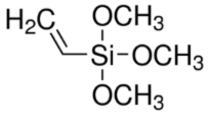
3-(glycidoxypropyl)trimethoxysilane<br>- U-50	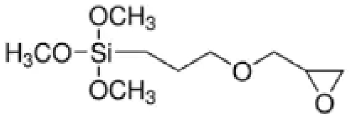
Vinyl-tris(2-methoxy-ethoxy)silane<br>- LUVOMAXX VTMOEO DL50 C	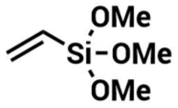
Mercaptopropyltrimethoxysilane<br>- Dynaslan MTMO	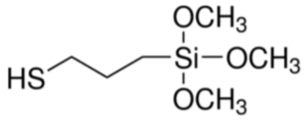
Bis(triethoxysilylpropyl)polysulfide<br>- Si-266	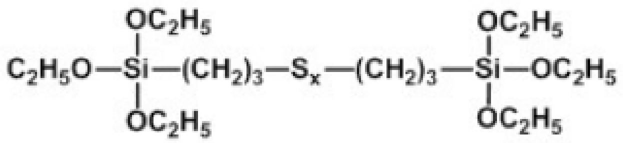

Table S2. Influence of thermal aging on hardness (H) of the rubber vulcanizates.

Parameter / Sample	H [˚ShA]	H after ageing [˚ShA]	ΔH [%]
CB+FA	71	72	+1.4
CB+FA-63	68	68	0.0
CB+FA-125	66	66	0.0
CB+FA-250	66	66	0.0
CB+FA-63-U-50	76	76	0.0
CB+FA-125-U-50	76	74	-2.7
CB+FA-250-U-50	75	74	-1.4
CB+FA-63-U-611	68	66	-3.0
CB+FA-125-U-611	67	66	-1.5
CB+FA-250-U-611	66	66	0.0
CB+FA-63-Si-266	77	75	-2.7
CB+FA-125-Si-266	76	76	0.0
FA-250-Si-266	74	75	+1.3
CB+FA-63-VTMOEO	67	66	-1.5
CB+FA-125-VTMOEO	65	66	+1.5
CB+FA-250-VTMOEO	65	65	0.0
CB+FA-63-MTMO	71	71	0.0
CB+FA-125-MTMO	70	70	0.0
CB+FA-250-MTMO	69	69	0.0

Studies were carried out according to PN-ISO 188:2000. in the KC-65 type thermal chamber. Rubber samples were aged at 70 °C, for 72 hrs.
